# A Building Extraction Method for High-Resolution Remote Sensing Images with Multiple Attentions and Parallel Encoders Combining Enhanced Spectral Information

**DOI:** 10.3390/s24031006

**Published:** 2024-02-04

**Authors:** Zhaojun Pang, Rongming Hu, Wu Zhu, Renyi Zhu, Yuxin Liao, Xiying Han

**Affiliations:** 1 School of Geomatics, Xi’an University of Science and Technology, Xi’an 710054, China; 2 School of Geological Engineering and Geomatics, Chang’an University, Xi’an 710054, China; zhuwu@chd.edu.cn; 3 The First Institute of Geoinformation Mapping, Ministry of Natural Resources, Xi’an 710054, China

**Keywords:** high-resolution remote sensing imagery, building extraction, deep convolutional neural network (DCNN), transformer, spectral enhancement

## Abstract

Accurately extracting pixel-level buildings from high-resolution remote sensing images is significant for various geographical information applications. Influenced by different natural, cultural, and social development levels, buildings may vary in shape and distribution, making it difficult for the network to maintain a stable segmentation effect of buildings in different areas of the image. In addition, the complex spectra of features in remote sensing images can affect the extracted details of multi-scale buildings in different ways. To this end, this study selects parts of Xi’an City, Shaanxi Province, China, as the study area. A parallel encoded building extraction network (MARS-Net) incorporating multiple attention mechanisms is proposed. MARS-Net builds its parallel encoder through DCNN and transformer to take advantage of their extraction of local and global features. According to the different depth positions of the network, coordinate attention (CA) and convolutional block attention module (CBAM) are introduced to bridge the encoder and decoder to retain richer spatial and semantic information during the encoding process, and adding the dense atrous spatial pyramid pooling (DenseASPP) captures multi-scale contextual information during the upsampling of the layers of the decoder. In addition, a spectral information enhancement module (SIEM) is designed in this study. SIEM further enhances building segmentation by blending and enhancing multi-band building information with relationships between bands. The experimental results show that MARS-Net performs better extraction results and obtains more effective enhancement after adding SIEM. The IoU on the self-built Xi’an and WHU building datasets are 87.53% and 89.62%, respectively, while the respective F1 scores are 93.34% and 94.52%.

## 1. Introduction

Buildings, as essential places for daily life and production for people, are crucial carriers for showcasing urban culture, history, and modernization levels. It is also an integral component of urban basic geographic information construction. At the same time, the reasonable layout of buildings has a crucial impact on urban development, environmental construction, and people’s lives. The construction, demolition, renovation, expansion, and other activities also reflect the potential for growth in a particular area [1,2,3]. Therefore, extracting accurate building data plays a crucial role in urban planning and development [4,5], land use change detection [6], national defense construction [7], disaster prevention and mitigation [8], environmental protection [9], and other aspects.

With the rapid development of high-resolution sensor technology and equipment, the acquisition of remote sensing images (RSIs) has become more flexible and efficient, and the spectral and spatial resolutions have been further improved. These mean richer information, sharper detail in the imagery, and a high-quality data source for more accurate building extractions [10]. However, extracting building information from high-resolution RSIs also brings more challenges to image interpretation. Due to the large amount of information and comprehensive coverage of high-resolution RSIs, as well as the diversity in building spectral characteristics, scale, morphology, etc., accurately and efficiently extracting buildings from high-resolution RSIs remains a vital research direction [11,12]. As a result, there is a need for more automated, accurate, and efficient image interpretation methods to match this, and the significant emergence of high-resolution remote sensing image data also leads to a shift towards a data-intensive scientific paradigm in earth observation research.

Deep learning can automatically extract the required features and uncover more in-depth information about objects. It is an inheritance and development of traditional machine learning techniques, bringing new solutions to the semantic segmentation tasks for building extraction in remote sensing images [13]. It is worth noting that classic deep learning networks, the U-shaped network (U-Net) [14] and the residual network (ResNet) [15], have achieved significant segmentation results. Inspired by their structures, many scholars have made network improvements based on them or simultaneously deepened the network structure to construct deep convolutional neural networks (DCNNs) for extracting deeper features of buildings. Jin et al. [16] combined the dense atrous spatial pyramid pooling (DenseASPP) [17] with the U-Net structure to build the bilateral attention refinement network (BARNet), which can refine the perception of building boundaries. Xu et al. [18] used ResNeXt101 to replace the encoder part of U-Net and combine it with a feature pyramid to fuse multi-scale features to improve the building segmentation accuracy for small sample datasets. Yu et al. [19] added a recurrent, residual deformable convolution unit based on the U-Net structure and blended in enhanced multi-head self-attention during the jump connection process to improve the network’s extraction details for complex buildings. Aryal et al. [20] incorporated multi-scale feature maps with a partial feature pyramid network into the U-Net framework to achieve higher precision and robustness in building extraction. DCNN has become the mainstream method for automatic building extraction [21]. Still, its small and fixed field of view, which focuses more on local context information, limits the deep learning network from extracting building features in complex backgrounds of high-resolution remote sensing images.

In recent years, the transformer architecture can capture global context information and long-range dependencies between pixels, providing new technical support for building extraction. Swin transformer [22] achieves attention operation and information sharing in a single window. Networks such as the segmentation transformer (SETR) [23], the semantic segmentation with transformers (SegFormer) [24], and the Unet-like pure transformer (Swin-Unet) [25] adopt a pure transformer structure and have achieved good segmentation results. However, while the transformer can capture global information, the advantage of local feature extraction via convolutional neural networks (CNNs) cannot be replaced entirely [26]. UNet-like transformer (UNetFormer) [27] uses a lightweight ResNet18 as the encoder, combined with a transformer-based decoder to extract global and local features. Zhang et al. [28] used the Swin transformer as the encoding structure. They constructed DCNN as the decoding structure to improve the segmentation effectiveness of the building boundaries in very high-resolution RSIs. Wang et al. [29] proposed a multiscale transformer with the convolutional block attention module (MTCNet), which combined the convolutional block attention module (CBAM) [30] and transformer to improve the network’s segmentation performance for buildings in RSIs. He et al. [31] proposed utilizing the Swin transformer to assist UNet (ST-UNet) for feature extraction in RSIs, which uses U-Net as the primary encoder and the Swin transformer as an auxiliary encoder. The network also uses a designed relational aggregation module (RAM) to guide the primary encoder with channel relationships, thereby improving the network’s global modeling capabilities. Li et al. [32] added multiple transposed convolution sampling modules in SegFormer to enhance the local and long-range detail on building information. Xia et al. [33] constructed a dual-stream feature extraction encoder based on ResNet34 [15] and Swin transformer. They performed feature aggregation at each network stage, effectively enhancing the network’s capability for building extraction. However, despite the many advantages of transformers in building extraction, their complexity still needs to be improved. Their performance may not be ideal in cases where the training dataset is small. Additionally, due to the complex background information in high-resolution RSIs, extracted buildings may be similar to and adjacent to surrounding roads, leading to the need for mitigation of false positives and false negatives. Therefore, further research on leveraging the advantages of DCNNs in local feature extraction and the global information-capturing capabilities of transformers is of significant importance for building extraction in high-resolution remote sensing images.

The effectiveness of deep learning networks largely depends on the diversity and quality of the dataset for effective building extraction. The differences in building forms and distributions across different regions further increase the difficulty of semantic segmentation tasks for buildings. Currently used open-source building datasets include the aerial image segmentation dataset [34], WHU building dataset [35], INRIA aerial image dataset [36], Massachusetts buildings dataset [37], etc. These building datasets were obtained through aerial photography and have high resolution, such as the 0.075 m resolution of the aerial image segmentation dataset and the 0.3 m resolution of the WHU building dataset. They have diverse architectural styles covering Europe, East Asia, the United States, and New Zealand. However, the current building dataset contains few images of architectural styles within China, and most are three-band images (red (R), green (G), and blue (B) datasets). Further exploration of the spectral information of the images is still needed to complement semantic segmentation networks for more accurate building extraction work.

To address the abovementioned issues further and improve the extraction performance of multi-scale buildings under different data sources, this paper explores combining transformer with DCNN and further mining the spectral information of high-resolution RSIs. Based on the U-Net architecture, we employed a parallel encoder of Swin transformer and ResNet to extract building feature information at various scales simultaneously using advantageous local and global extraction methods. To enhance the recognition capability of local blurry features in remote sensing images, we introduced the DenseASPP module in the decoding process and combined it with the feature information obtained from skip connections. Various attention mechanisms were also introduced in the skip connection process at different stages of the network to capture and retain the positional and semantic feature information of the downsampled feature maps. In addition, this study also constructed the SIEM module to enhance the spectral information of RSIs and further improve the accuracy of building extraction. We selected part of Xi’an City, Shaanxi Province, China, as the study area and used Gaofen-2 satellite (GF-2) images for the Xi’an building dataset. The study area contains buildings of various forms, distributions, and scales, providing a dataset of buildings with the characteristic architectural style of Xi’an. The dataset includes images with R, G, B, and near-infrared (NIR) bands, which can provide data support for various spectral processing methods.

The contributions of this paper mainly include the following aspects:

(1) We used high-resolution satellite images from GF-2 to construct the Xi’an building dataset, which includes complex background information and features various building forms, distributions, and scales. This dataset enriches the diversity of datasets used for building extraction and presents more challenges for building extraction networks.
(2) We designed an effective building extraction network, MARS-Net, to improve the extraction performance of buildings with different architectural characteristics in different regions. We compared MARS-Net with other building extraction methods on our self-built Xi’an building dataset and the WHU building dataset, and conducted ablation experiments, demonstrating the effectiveness and generalization of the proposed network in this study.
(3) Using the Xi’an building dataset, we propose a spectral information enhancement module to enhance the relationships between bands of high-resolution remote sensing images and provide them with reinforced building shape information. Through experiments, it is demonstrated that this module can effectively enhance the extraction of buildings in complex backgrounds by semantic segmentation models.

The rest of this paper is organized as follows: Section 2 mainly introduces the design of the proposed method. Section 3 presents the building dataset constructed for this study, the comparative datasets used in the experiments, the primary parameter settings, and the selection of evaluation metrics. It reports the results of the network’s comparative and ablation experiments and conducts experimental analysis of the SIEM module after validating the effectiveness of the network. Section 4 discusses the work carried out in this paper. Section 5 summarizes the entire paper and looks forward to future work.

## 2. Materials and Methods

### 2.1. Network Architecture Overview

**Figure 1:**
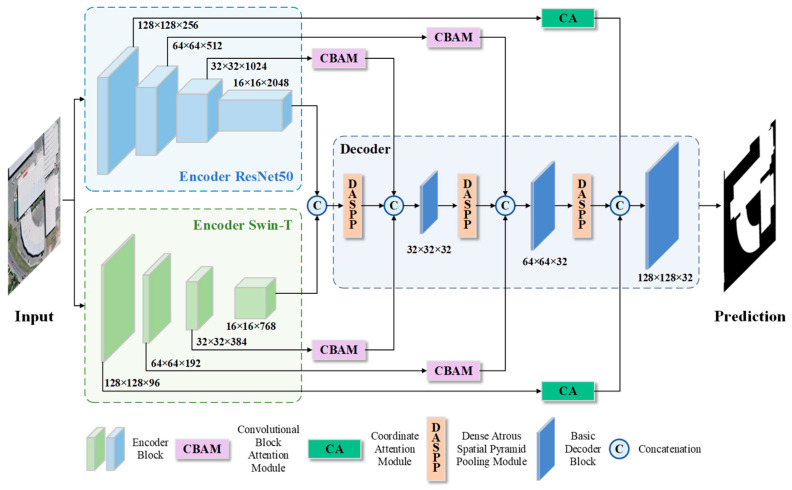
The architecture of MARS-Net overview architecture.

Multi-scale fusion analysis refers to the high-level network acquiring detailed information in the image and the low-level network obtaining deep-layer information [38]. Therefore, when constructing the network, considering different perspectives in the structure to integrate multi-scale details of the image can enable better recognition of target information in remote sensing images. Based on this, we propose MARS-Net, as shown in Figure 1.

The network comprises an encoder, decoder, and skip-connection parts, combined with DCNN, transformer, and attention mechanism to collect context information of different scales in various network parts, enhancing the ability to learn building features. The encoder part consists of a parallel backbone structure of ResNet50 [15] and Swin-T (Tiny) [22], which collectively extract shallow features of buildings. The introduction of the DenseASPP module in the decoder obtained a larger receptive field, allowing the network to acquire semantic feature information densely over a more extensive scale range. Adding skip connections between the encoder and decoder. Incorporating coordinate attention (CA) [39] in shallow-level skip connections to capture more spatial location features. Using the CBAM module in deep-level skip connections to capture more semantic features achieves a balance between feature channel and spatial dimensions.

#### 2.1.1. Parallel Encoding Architecture

The encoding part of MARS-Net consists of a parallel encoder with ResNet50 and Swin-T backbone networks. The ResNet50 backbone leverages the advantages of DCNN for extracting features of small and dense buildings. In contrast, the Swin-T backbone uses its capability to capture global contextual features to enhance the network’s ability to obtain building feature information in different backgrounds.

**Figure 2:**
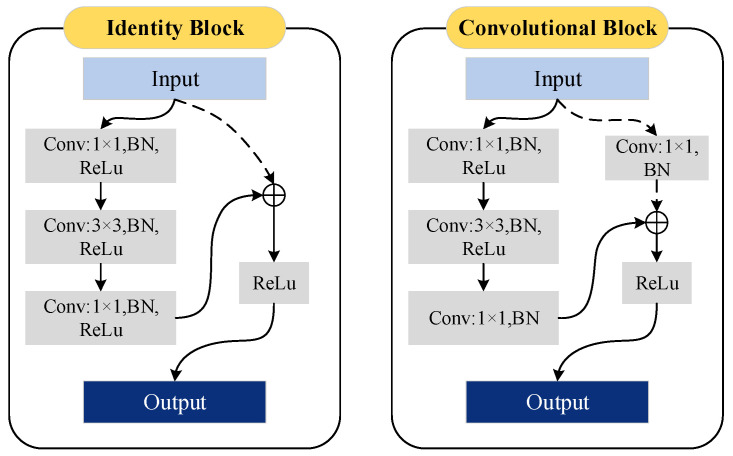
Residual blocks. On the left is the identity block structure, and on the right is the convolutional block structure. They utilize batch normalization (BN) to expedite training and employ the ReLU activation function. The dashed line connections were dimensionally processed using 1 × 1 convolutional kernels. The solid arrow is the standard feature information transfer process. The dashed arrows indicate that the transfer process is the shortcut connection process, which enables the cross-layer transfer of feature information.

The ResNet50 backbone network in MARS-Net is shown on the left in Figure 1. First, the feature maps will pass through a 7 × 7 convolutional layer and a 3 × 3 max pooling layer. The 7 × 7 kernel size is set to increase the receptive field, preserving global information and semantic associations with neighboring features at the input layer to the network, as well as establishing complex spatial relationships, thereby improving network performance. The following four stages are each composed of residual blocks, as depicted by the structure of submodules shown in Figure 2. The ResNet50 uses residual blocks with the following structure numbers: 3, 4, 6, and 3, respectively. It also uses multiple convolutional kernels for feature extraction, providing high flexibility [15]. The direct flow of information between layers enhances the network’s representative capability, enabling the construction of deeper DCNN networks that can better extract local architectural details.

**Figure 3:**
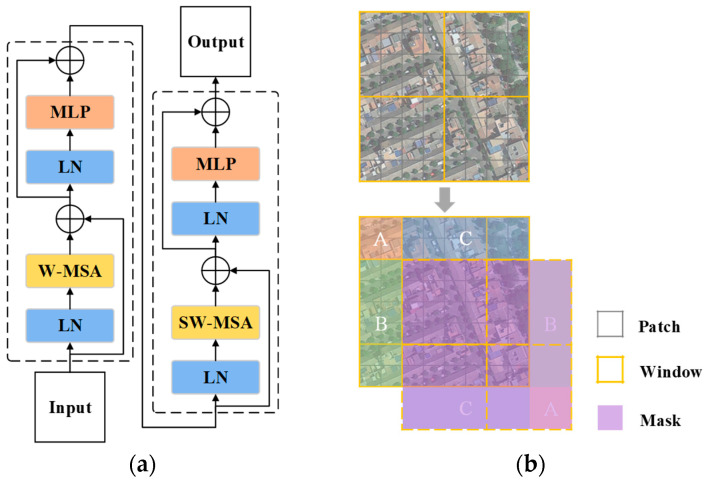
Swin transformer basic architecture and operation schematics. (**a**) Swin transformer block; (**b**) W-MSA and SW-MSA operation schematics. Those areas marked A–C are the shifted windows used to SW-MSA.

The Swin-T encoder in MARS-Net is shown on the left side in Figure 1. The input feature map is first divided into adjacent pixels and flattened in the channel direction by the patch partition layer. Then, each pixel’s channel data are linearly transformed by the linear embedding layer, followed by obtaining new feature representations through a fully connected layer. In the following three parts, the patch merging layer is used for down-sampling and preventing loss of feature information by concatenating pixels. The Swin transformer block is shown in Figure 3a. Under the Swin-T configuration, there are six sets in Stage 3 and two sets in the other Stages.

The computational process of the window multi-head self-attention (W-MSA) and shifted window multi-head self-attention (SW-MSA) introduced in the Swin transformer block is shown in Figure 3b. In W-MSA, the feature map in Figure 3b is divided into 2 × 2 windows, and independent self-attention calculations are performed in each patch. SW-MSA shifts the window of W-MSA to the bottom right corner by two units to obtain nine new windows. Then, the orange window block A, the green window block B, and the blue window block C in Figure 3b are moved to the bottom right corner of the dashed box to obtain new 2 × 2 windows. To avoid the issue of feature information disorder caused by window shifting, a masked MSA is used for masking operations, as shown in the purple area in Figure 3b, to isolate information from different areas and enhance the ability of blocks to extract global features within the image.

#### 2.1.2. Dense Atrous Spatial Pyramid Pooling Module

Due to the more complex background information in high-resolution RSIs and the increased depth of feature extraction in the network encoder, the original U-Net decoder cannot effectively meet the requirements for feature information upsampling and concatenation. Also, it fails to capture the detailed boundary information of the buildings fully. This study incorporates a DenseASPP structure into the feature decoding part to capture and integrate multi-scale contextual information during upsampling. This improves the network’s multi-scale building edge feature extraction.

**Figure 4:**
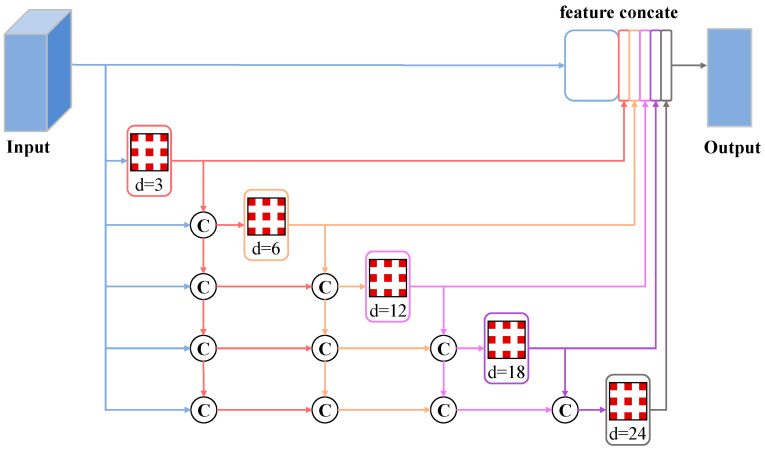
The architecture of dense atrous spatial pyramid pooling module. Different levels of module processing are distinguished by different colors. The symbol “C” represents the concatenation operation of the feature map. The checkerboard rectangle represents the atrous convolution operation. “d” represents the expansion rate.

Atrous spatial pyramid pooling (ASPP) gathers pixel-level feature information through different grid scales and sampling rates [40]. Significant dilation rates are needed to achieve a larger receptive field in ASPP. However, as the dilation rates increase, the convolution will gradually become ineffective, weakening the recovery effect of building feature information [41]. In this study, the DenseASPP is used as the pooling structure before concatenating with the skip-connected feature information, aiming to restore and generate dense building features over a larger area during the upsampling process, as shown in the decoding section on the right side of Figure 1. The final output feature map of DenseASPP covers semantic feature information not only over a large scale, but also in a very dense manner. Due to the varying degrees of spatial and semantic information loss at different depths in the feature maps generated by the decoder, this study selects dilation rates (d) of 3, 6, 12, 18, and 24 for the five different atrous convolutional layers. The output features of each convolution layer are spliced together in parallel and cascade to recover the lost spatial and semantic information of the building, as shown in Figure 4.

#### 2.1.3. Coordinate Attention

**Figure 5:**
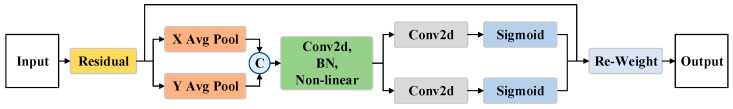
The architecture of coordinate attention. The input feature maps are first average-pooled along the X and Y directions separately, then concatenated. The resulting feature map undergoes batch normalization (BN) and non-linear regression and is then split. Finally, the attention vector is obtained by combining it with a sigmoid activation function. The symbol “C” represents the concatenation operation of the feature map.

Coordinate attention (CA) uses two one-dimensional global pooling operations to aggregate the input features in the vertical and horizontal directions independently, resulting in two separate direction-aware feature maps. These two feature maps embedding specific directional information are encoded as two attention maps. Each captures long-range dependencies along a spatial direction of the input feature maps. As a result, the location information is held in the generated attention map. The two attention maps are combined into the input feature map to enhance the symbolic power of the feature map [39]. Figure 5 shows the structure of CA.

As the network layers deepen, the semantic feature information of the feature maps will continuously increase, while the spatial positional feature information of the feature maps will gradually diminish. Therefore, we designed this module as skip connections in the first layer, which has more spatial data, to collect more spatial feature information during the downsampling process and apply it to the upsampling process to reduce the loss of local semantic feature details in the image.

#### 2.1.4. Convolutional Block Attention Module

**Figure 6:**
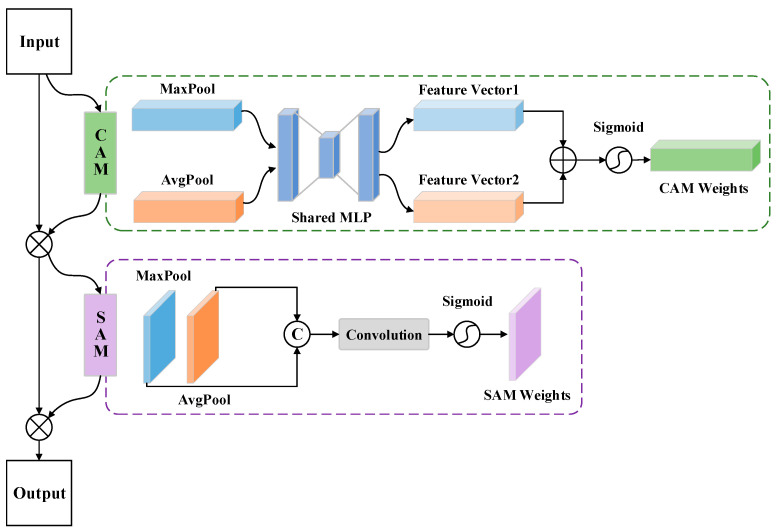
The architecture of the convolutional block attention module. The symbol “C” represents the concatenation operation of the feature map.

The CBAM module comprises two sub-attention modules: the channel attention module (CAM) and the spatial attention module (SAM), which pay attention to the channel and spatial dimensions, respectively. Since the middle and deeper layers of the network contain rich spatial information and semantic feature information, we designed skip connections in the form of the network at the second and third layers, as shown in Figure 1. Such a structure enables CBAM to compute weights with the help of assignments in the channel and spatial dimensions sequentially, thus placing more emphasis on building target features [30]. While weakening the influence of background data, it improves the building prediction ability of the network and guarantees the stability of the model operation. CBAM first applies global max pooling and global average pooling operations to the surface feature data in the channel dimension. Then, using a fully connected layer, it assigns weight to the feature vector, enhancing the feature information in the channel dimension. Afterward, in the spatial dimension, the feature vector obtained from the channel domain is subjected to max pooling and average pooling compression to obtain a two-dimensional feature vector. Finally, convolution is used to assign weights to enhance the feature information of the building. Figure 6 shows the architecture of CBAM.

#### 2.1.5. Loss Function

This paper uses the Dice loss [42] as the network’s loss function. It measures the accuracy of the prediction by calculating the overlap between the segmented pixels and the labeled pixels, thus assisting the model in coping with the imbalance between the number of buildings and background pixels in the image. The formula of Dice loss is defined as:

(1) $$L_{dice}=1-\frac{2\left|Pred\cap GroundTruth\right|}{\left|Pred\right|+\left|GroundTruth\right|},$$

where *Pred* represents the predicted set of building pixels, and *Ground Truth* represents the set of pixels for building labels.

### 2.2. Spectral Information Enhancement Module

#### 2.2.1. Module Architecture Overview

**Figure 7:**
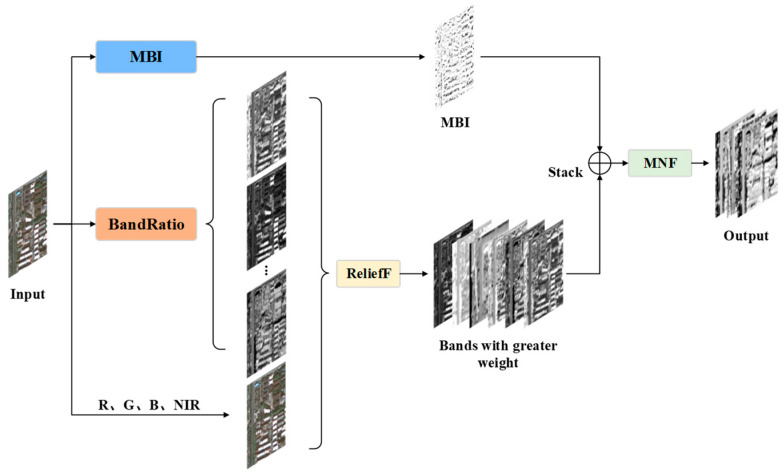
The architecture of the spectral information enhancement module.

High-resolution RSIs have a large amount of information and possess high spectral and spatial resolution. To further explore the spectral information and improve the segmentation effect of buildings in the network, we propose SIEM inspired by [43], as shown in Figure 7. This module uses the R, G, B, and NIR bands of high-resolution RSIs to calculate the morphological building index (MBI) and band ratios. Then, it performs the ReliefF operation on the band ratios, R, G, B, and NIR, to select the six bands with the highest weights. SIEM stacks the screening results with MBI, and the stacked images are then fused using maximum noise fraction (MNF) operation for dimensionality reduction. In the end, we can obtain an enhanced image with more emphasis on the band relationship and the morphological information of the buildings.

#### 2.2.2. Spectral Information Expansion Combined with Near-Infrared Band Ratios

The imagery typically input into a deep learning network is divided into the R, G, and B bands, and convolution operates using the pixels and surrounding information within the same band. It is only after addition that information from other bands is involved. Therefore, only single-band information is sensed during the convolution process without emphasizing the interrelationships between bands. In response to this issue, [43] proposed a red–blue band ratio enhancement operation based on normalized vegetation index to enhance the computer’s understanding of relationships between bands. However, this method only involves the relationship between red and blue bands. To further emphasize the inter-band relationships and improve the network’s recognition of semantic features of buildings, we propose a ratio-weighted method for R, G, B, and NIR based on our self-built Xi’an dataset and its characteristics in the near-infrared band. This method involves pairwise ratio operations among these four bands, followed by using the ReliefF [44] feature selection algorithm to assign different weights to the features and select six bands with higher weight coefficients for synthesizing spectral information between multi-band images. The calculation for the inter-band ratio is as follows:

(2) $$G_{mn}(i,j)=0.5\times (\frac{b_{m}(i,j)-b_{n}(i,j)}{b_{m}(i,j)+b_{n}(i,j)})+0.5,$$

where (*i*, *j*) represents the coordinates of the pixel point in the image, *mn* represents the combination of two different bands, *G_mn_*(*i*, *j*) represents the ratio of band *m* and band *n* at point (*i*, *j*), and *b_m_*(*i*, *j*) represents the value at point (*i*, *j*) on band *m*. The coefficients and constant term are set to 0.5 to ensure that the range of values is between 0 and 1.

#### 2.2.3. Spectral Information Enhancement Based on Morphological Building Index

Using morphological building index (MBI) with R, G, B, and NIR image data results in better performance than using only R, G, and B band image data [45]. Accordingly, we calculated the MBI on our self-built Xi’an building dataset and concatenated it with several ratio bands with larger weights selected by ReliefF. Then, we used the MNF algorithm to fuse and reduce the dimensionality of the concatenated image. This allows each band to have rich spectral information while having more robust building morphology characteristics, thereby enhancing the spectral information of the image and improving the effectiveness of network segmentation of buildings.

The calculation method of MBI is typically based on the grayscale levels or color information in the image, combined with morphological operations (such as erosion, dilation, opening, closing, etc.) to extract the morphological features of buildings. First, calculate the brightness value of the image. The spectral band’s maximum value represents the feature’s high reflectance characteristics. So, the brightness value is taken as the maximum value of each pixel in all bands, which is defined as follows:

(3) $$b\left(x\right)=\underset{1\leq i\leq K}{\max}\left(band_{i}(x)\right),$$

where *band_i_*(*x*) represents the spectral value of pixel *x* in the *i* band, *b*(*x*) is the brightness, and *K* is the total number of bands. Then, morphological white top-hat reconstruction is performed, which first uses the structuring element *s* to perform opening operation on the image *b*, which is expressed as follows:

(4) $$\gamma^{s}(b)=\delta^{s}(\varepsilon^{s}(b)),$$

where *γ^S^*(*b*) represents the result of the opening operation, *ε* denotes the erosion operation, *δ* indicates the dilation operation, and *s* represents the size of the structuring element (i.e., the size of the convolutional kernel). The white top-hat transformation is performed on the result of the opening operation, which is defined as follows:

(5) $${\mathrm{THR}}^{s}(b)=b-\gamma_{RE}^{s}(b),$$

where THR stands for top-hat by reconstruction, and *γ_RE_* represents opening by reconstruction. THR reflects the brightness differences within the structural element region and between adjacent objects, so the features related to structural contrast are included in the THR feature. In directional aspects, MBI distinguishes from the road by detecting objects’ anisotropy and linear structural elements in multiple directions. It focuses on extracting buildings through multi-directional morphological scale reconstruction with the following formula:

(6) $${\bar{\mathrm{THR}}}^{s}(b)=\underset{dir}{{\mathrm{mean}(\mathrm{THR}}^{s.dir}(b))},$$

where *dir* represents the directional nature of the structuring element, and the average value represents the multi-directional information of THR. Multi-scale information of the image is extracted using the granulometry determination method of the white-top reconstruction, with the following formula:

(7) $$\left\{ \begin{array}{c} {\mathrm{THR}}_{\mathrm{DMP}}=\left\{{\mathrm{THR}}_{\mathrm{DMP}}^{S^{\min}},\cdot \cdot \cdot ,{\mathrm{THR}}_{\mathrm{DMP}}^{S},\cdot \cdot \cdot ,{\mathrm{THR}}_{\mathrm{DMP}}^{S^{\max}}\right\}\\ {\mathrm{THR}}_{\mathrm{DMP}}^{S}=\left|{\bar{\mathrm{THR}}}^{S+\Delta S}(b)-{\bar{\mathrm{THR}}}^{S}(b)\right|\\ S^{\min}\leq S\leq S^{\max} \end{array}\right.,$$

where Δ*S* represents the interval of particle determination, THR_DMP_ stands for multi-scale THR (i.e., a collection of differential morphological profiles (DMP)), and THR*^S^*_DMP_ represents the differential morphological profile with parameter *S*. With the above calculations, the brightness, contrast, directionality, and size characteristics of the image are processed separately. Thus, the MBI value can be obtained by averaging the four implicit features of buildings contained in THR_DMP_, which is defined as follows:

(8) $$\mathrm{MBI}=\underset{s}{\mathrm{mean}}({\mathrm{THR}}_{\mathrm{DMP}})$$

## 3. Experiments and Results

### 3.1. Dataset Details

#### 3.1.1. Study Area

**Figure 8:**
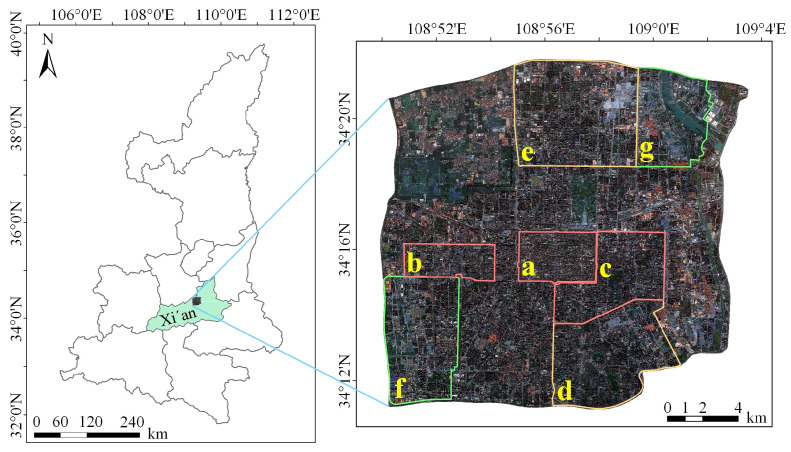
Study area. The red, orange, and green boxes on the right image indicate that the distribution of buildings in the area is dense, denser, and sparser, respectively. Those marked a–c are densely building areas; d and e are mid-density building areas; f and g are sparse building areas.

**Figure 9:**
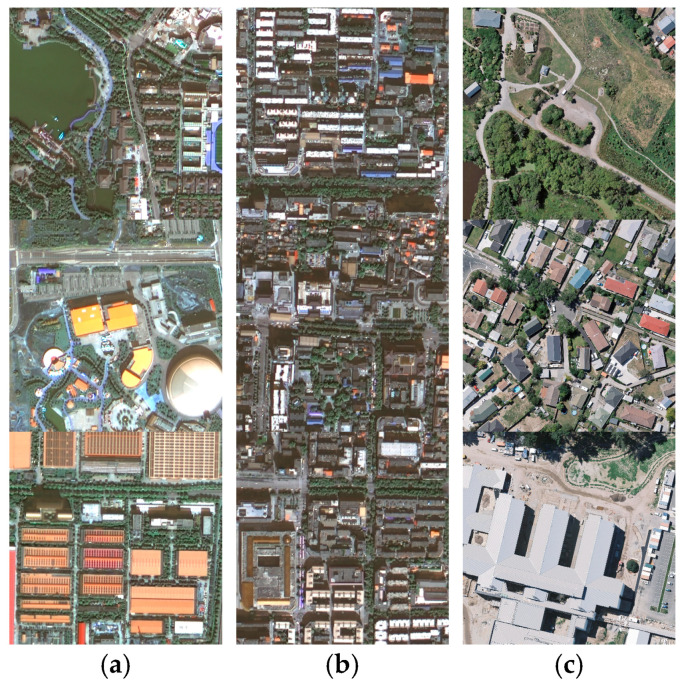
Examples of building forms, scales, and distributions in the Xi’an building dataset and the WHU building dataset: (**a**) buildings with different scales, forms, and spectral characteristics in the study area; (**b**) multi-scale buildings densely distributed in the study area, similar to the complex streets around them; (**c**) typical buildings in the WHU dataset.

Based on the high-resolution RSIs from China’s GF-2 within the third ring road of Xi’an, Shaanxi Province, China, as shown in Figure 8, we delineated seven areas in sequence based on the density of buildings (areas a–g in Figure 8) for the study. Xi’an is located in the Guanzhong Basin in the central part of the Yellow River basin in China, and it has a warm temperate, semi-humid continental monsoon climate. Due to its large population and rapid development, Xi’an exhibits a higher density of buildings in the study area than the WHU building dataset. The buildings vary in scale, have complex forms, and are primarily long rectangles or squares. Similarities and adjacency between roads and buildings are also common, as shown in Figure 9. The complex background information, multi-scale building morphology, and dense building distribution characteristics in the study area have placed higher demands on the extraction accuracy and generalization of building extraction algorithms.

The images in the Xi’an building dataset have a cloud coverage of 0 with a spatial resolution of 0.8 m panchromatic and 3.2 m multispectral, obtaining an image with a spatial resolution of 1 m after image preprocessing, stitching, cropping, mosaicing, and fusion operations. We processed the images into the R, G, and B three-band images while also generating four-band images containing R, G, B, and NIR for the subsequent testing experiments with the SIME module. The Xi’an building dataset covers an area of approximately 163 km^2^ and includes labels for over 35,000 buildings. We used the sliding window method; images and labels were cropped at a 0.35 overlap rate and a size of 512 × 512, resulting in 1662 images and labels. They will also expand to 8310 sheets through data enhancement operations such as pretzel noise, left–right flip, up–down flip, and diagonal mirroring. The Xi’an building dataset consists of 5567 images for training, 1413 images for validation, and 1330 images for testing. The training, validation, and test sets all contain urban buildings of varying densities in study areas a–g.

#### 3.1.2. WHU Building Datasets

To further analyze the network’s performance and validate the building dataset’s quality and reliability. We also used the WHU Aerial Building Imagery dataset. The WHU building dataset was proposed in 2018, which is more recent in time, and the WHU dataset with high image resolution and labeling accuracy has been used by many teams in building segmentation experiments in recent years [46,47,48]. Therefore, this study chose the WHU building dataset as the public dataset. This dataset consists of aerial imagery from Christchurch, New Zealand, with an original spatial resolution of 0.3 m, including R, G, and B channels. It covers an area of 450 km^2^ and contains approximately 22,000 individual buildings. It has 8189 images of 512 × 512 pixels, of which 4736 images are for the training set, 2417 images for the test set, and 1036 images for the validation set.

### 3.2. Experimental Settings and Evaluation Indicators

All experiments in this research were conducted on a Linux operating system, using an NVIDIA GeForce RTX 3090 GPU with 24 GB of memory, programmed in Python 3.7. The deep learning framework used was PyTorch 1.8.1, and the GPU computing platform was CUDA 11.1. The network used the Adam optimizer [49] with an initial learning rate of 0.0001. The models were trained with six images per batch and 100 epochs.

To test the effectiveness and accuracy of the various networks, the experiment selected six metrics, including the overall accuracy (OA), Kappa coefficient (Kappa), intersection over union (IoU), recall, precision, and F1 score, to evaluate the networks‘ classification performance. OA reflects the proportion of correctly classified pixels to the total pixels. At the same time, the Kappa coefficient, which is further calculated based on OA, is used to examine the consistency between the network‘s predicted results and the actual label results. IoU is the ratio of the intersection area of the predicted result and the ground truth annotation to the union area. Recall measures whether there are any omissions in the results. Precision measures whether there are any false positives in the results. The F1 score can consider the classification results’ precision and recall. The calculations for each indicator are defined as follows:

(9) $$\mathrm{OA}=\frac{\mathrm{TP}+\mathrm{TN}}{\mathrm{TP}+\mathrm{TN}+\mathrm{FP}+\mathrm{FN}},$$

(10) $$\mathrm{Kappa}=\frac{\mathrm{OA}-P_{e}}{1-P_{e}},$$

(11) $$P_{e}=\frac{\left(\mathrm{TP}+\mathrm{FP}\right)\times \left(\mathrm{TP}+\mathrm{FN})+(\mathrm{TN}+\mathrm{FN}\right)\times \left(\mathrm{TN}+\mathrm{FP}\right)}{{\left(\mathrm{TP}+\mathrm{TN}+\mathrm{FN}+\mathrm{FP}\right)}^{2}},$$

(12) $$\mathrm{IoU}=\frac{\mathrm{TP}}{\mathrm{TP}+\mathrm{FP}+\mathrm{FN}},$$

(13) $$\mathrm{Recall}=\frac{\mathrm{TP}}{\mathrm{TP}+\mathrm{FN}},$$

(14) $$\mathrm{Precision}=\frac{\mathrm{TP}}{\mathrm{TP}+\mathrm{FP}},$$

(15) $$F1=\frac{2(\mathrm{Precision}\times \mathrm{Recall})}{\mathrm{Precision}+\mathrm{Recall}},$$

where TP represents the number of correctly classified building pixels, FP represents the number of falsely detected building pixels, TN represents the number of correctly classified background pixels, and FN represents the number of missed building pixels.

### 3.3. Network Experimental Results and Analysis

To evaluate the performance of the MARS-Net proposed by us in the building semantic segmentation task, we conducted comparative experiments and ablation experiments on the WHU building dataset, which, like our Xi’an building dataset, consists of images with R, G, and B three bands. The comparative experiments compared MARS-Net with several mainstream DCNN and transformer networks, including U-Net, ResUNet++ [50], Deeplab v3+ [51], Swin-Unet, and UNetFormer.

#### 3.3.1. Comparative Experiments on the Xi’an Building

**Figure 10:**
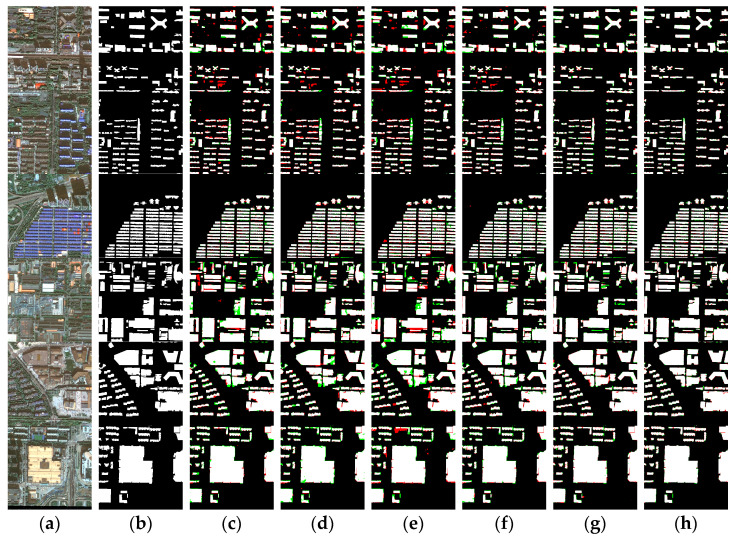
Visual comparison of results on the Xi’an building dataset: (**a**) original images; (**b**) ground truth; (**c**) U-Net; (**d**) ResUNet++; (**e**) Deeplab v3+; (**f**) Swin-Unet; (**g**) UNetFormer; (**h**) MARS-Net. Colors assigned: TP in white, TN in black, FP in red, and FN in green.

Figure 10 presents the visualized prediction results of different networks on the Xi’an building dataset, including various distribution densities and different sizes of buildings. Figure 10 shows that U-Net, ResUNet++, Deeplab v3+, and Swin-Unet have extracted most of the buildings. However, there are still occurrences of both false positives and false negatives (indicated in blue and red, respectively). On the other hand, UNetFormer and our proposed MARS-Net achieved more satisfactory results, and our network performs better in extracting buildings’ boundary and internal information. In the first and second rows, due to the similar spectral characteristics between buildings and the background, the recognition performance of the U-Net, ResUNet++, Deeplab v3+, and Swin-Unet DCNN networks is not ideal, with significant misclassification. UNetFormer can extract the shape information of buildings well, but the extraction results occasionally show noise and blurry boundary information. In the third row, the significant difference in spectral characteristics between buildings and the background allows each network to have good segmentation results. However, due to the small and dense nature of the buildings, there are certain missing or sticking issues with the extracted building boundaries.

Overall, the extraction results of the MARS-Net network are closest to the ground truth, and it performs better in extracting building targets of multiple scales and distribution forms. From the results in the fourth to sixth rows, U-Net, ResUNet++, and Deeplab v3+ perform relatively well in classifying small-sized buildings, but their performance is not ideal for classifying large and long-bordered buildings. On the other hand, Swin-Unet, UNetFormer, and MARS-Net all perform well in the extraction results. In addition, the issue of missing internal information in the extraction of buildings by MARS-Net has been further improved, and the boundary information is smoother. This is because our network combines the advantages of DCNN and transformer, which enhances the network‘s ability to capture long-distance boundary information of large buildings. Additionally, through the CA, CBAM, and DenseASPP modules carefully introduced at multiple stages of the network, MARS-Net effectively connects cross-layer information while retaining multi-scale contextual feature maps from different stages of the network. It allows MARS-Net to capture complete interior information of small buildings, improve boundary adhesion and false detection phenomena, and better overcome interference from similar spectral features.

Quantitative evaluation of the segmentation results for the Xi’an building dataset by different networks is presented in Table 1. In Table 1, the proposed MARS-Net network has 97.97% OA, 92.15% Kappa, 87.52% IoU, 93.67% recall, 93.02% precision, and 93.34% F1 score on the Xi’an building dataset. All evaluation metrics of this network show excellent performance. The first to fifth rows show the semantic segmentation accuracy evaluation results of the U-Net, ResUNet++, Deeplab v3+, Swin-Unet, and UNetFormer comparative networks. Compared to the U-Net, ResUNet++, Deeplab v3+, Swin-Unet, and UNetFormer algorithms, the OA respectively increased by 0.94%, 1.19%, 2.05%, 0.86%, and 0.61%; the Kappa respectively increased by 3.66%, 4.59%, 7.77%, 3.37%, and 2.36%; the IoU respectively increased by 5.32%, 6.59%, 10.86%, 4.90%, and 3.46%; the Recall respectively increased by 3.67%, 3.82%, 5.74%, 3.31%, and 2.23%; the Precision respectively increased by 2.55%, 3.95%, 7.35%, 2.42%, and 1.78%; the F1 score respectively increased by 3.11%, 3.88%, 6.56%, 2.86%, and 2.01%.

#### 3.3.2. Comparative Experiments on the WHU Building

**Figure 11:**
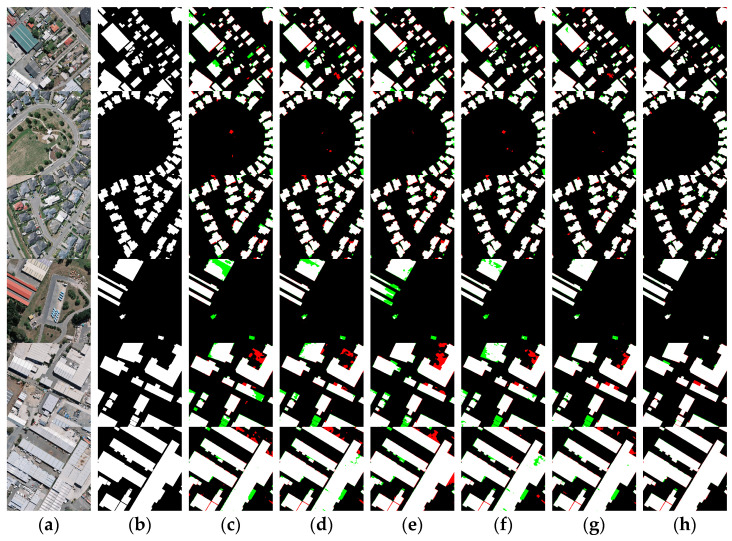
Visual comparison of results on the WHU building dataset: (**a**) original images; (**b**) ground truth; (**c**) U-Net; (**d**) ResUNet++; (**e**) Deeplab v3+; (**f**) Swin-Unet; (**g**) UNetFormer; (**h**) MARS-Net. Colors assigned: TP in white, TN in black, FP in red, and FN in green.

To verify the generalization of MARS-Net, we conducted the same comparative experiment on the WHU building dataset, as shown in Figure 11. Figure 11 shows that, compared with other advanced networks, the proposed MARS-Net further improves predicting results on various distributions and building sizes, significantly reducing misclassification and misses detections.

Specifically, in the predicted results of small buildings with different density levels from the first to the second row in Figure 11, U-Net, ResUNet++, Deeplab v3+, and Swin-Unet show poor edge integrity in their predictions. Although both UNetFormer and MARS-Net achieved good prediction results, MARS-Net retained more edge information on the buildings and improved in reducing false detections of small buildings. In addition, due to insufficient feature extraction capability and inadequate receptive field size of the encoder part of U-Net, ResUNet++, Deeplab v3+, and Swin-Unet networks, there are many holes in the extraction results when predicting large buildings, as shown in rows three to six of Figure 11. The phenomenon of holes in the extraction of large buildings by UNetFormer has been significantly improved. However, some misclassification of boundary information for large buildings still exists, and the extraction results for surrounding small buildings are less than ideal. The proposed network benefits from constructing a dual encoder comprising DCNN and transformer. And the introduction of CA and CBAM at different stages of the encoder to bridge the gap between the encoder and decoder. Thus, the two advantageous extraction methods preserve more building feature information. Adding the DenseASPP module can capture more contextual semantic details of buildings, thereby enhancing the network’s ability to resist interference from complex background information. Therefore, MARS-Net can extract buildings of different distributions and scales in complex backgrounds, making the internal information of large buildings more complete and the boundaries smoother. Meanwhile, it can also predict many small buildings with more precise outlines. While it enhances the extraction of large buildings, it also has good segmentation capabilities for small buildings.

The quantitative evaluation of the segmentation results of the WHU building dataset by different networks is shown in Table 2. As shown in Table 2, the proposed MARS-Net network has 98.78% OA, 93.84% Kappa, 89.62% IoU, 94.64% Recall, 94.41% Precision, and 94.52% F1 score on the WHU building dataset. All the evaluation metrics of this network show good performance. The first to fifth rows show the semantic segmentation accuracy evaluation results of the U-Net, ResUNet++, Deeplab v3+, Swin-Unet, and UNetFormer comparative networks. Compared to the U-Net, ResUNet++, Deeplab v3+, Swin-Unet, and UNetFormer algorithms, the OA has improved by 1.12%, 0.86%, 0.85%, 0.56%, and 0.49%; the Kappa has been enhanced by 5.60%, 4.25%, 4.34%, 2.87%, and 2.32%; the IoU has improved by 8.52%, 6.53%, 6.69%, 4.49%, and 3.61%; the recall has improved by 4.25%, 2.74%, 4.39%, 3.12%, and 1.47%; the precision has improved by 5.66%, 4.77%, 3.32%, 1.99%, and 2.61%; and the F1 score has increased by 4.96%, 3.76%, 3.86%, 2.56%, and 2.04%, respectively.

#### 3.3.3. Ablation Study

**Figure 12:**
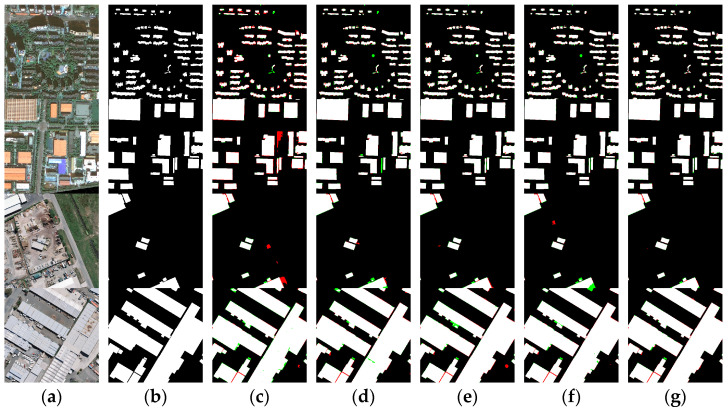
Results of ablation study on building dataset: (**a**) original images; (**b**) ground truth; (**c**) Baseline; (**d**) Baseline + CA; (**e**) Baseline + CBAM; (**f**) Baseline + DenseASPP; (**g**) MARS-Net. Colors assigned: TP in white, TN in black, FP in red, and FN in green. Segmentation results of the Xi’an building dataset for rows 1~2. Segmentation results of the WHU building dataset for rows 3~4.

In this section, we conducted ablation experiments on the Xi’an building dataset and the WHU building dataset to explore the effect of each module on the parallel coding structure of DCNN and transformer in MARS-Net. We used MARS-Net with only DCNN and transformer dual coding structure as the baseline network (Baseline). We separately added CA, CBAM, and DenseASPP to the Baseline, and the visualization results of the segmentation are shown in Figure 12. The quantitative evaluation results are shown in Table 3 and Table 4.

As shown in Figure 12, adding each module has improved the segmentation results compared to the baseline network. There is a significant improvement in the misidentification and missed detection of building boundaries, and it has reduced the occurrence of voids in the extraction results of large buildings. The spectral characteristics of buildings in the third and fourth rows are more similar to land cover features. However, introducing the three modules has improved the smoothness of boundary prediction for buildings based on the baseline network. The overall prediction results of MARS-Net have been further enhanced, with the internal information of buildings of different distributions and scales being complete. The boundaries are smoother and better at suppressing interference from similar background information.

As shown in Table 3, compared to the baseline network, the introduction of the CA module resulted in improvements of 0.77%, 2.11%, 2.76%, 3.26%, and 1.62% in OA, Kappa, IoU, recall, precision, and F1 score, respectively. The addition of the CBAM module resulted in improvements of 0.97%, 2.92%, 3.97%, 0.05%, 4.44%, and 2.31% in OA, Kappa, IoU, recall, precision, and F1 score, respectively. Meanwhile, adding the DenseASPP module led to respective improvements of 1.01%, 3.06%, 4.18%, 0.42%, 4.31%, and 2.43%. In the accuracy evaluation metrics of the WHU building dataset in Table 4, compared to the baseline network, adding the CA module led to improvements of 0.25%, 1.45%, 2.31%, 2.39%, 0.21%, and 1.31% in OA, Kappa, IoU, recall, precision, and F1 score, respectively, while adding the CBAM module led to respective improvements of 0.28%, 1.57%, 2.49%, 2.29%, 0.53%, and 1.42%, and the addition of the DenseASPP module resulted in respective improvements of 0.31%, 1.72%, 2.73%, 2.79%, 0.30%, and 1.55%, respectively. The performance of the baseline network can be improved overall when using the CA, CBAM, and DenseASPP modules on both datasets. Moreover, when combining all three modules, the network’s various accuracy evaluation metrics showed the largest overall improvement.

### 3.4. SIEM Experimental Results and Analysis

**Figure 13:**
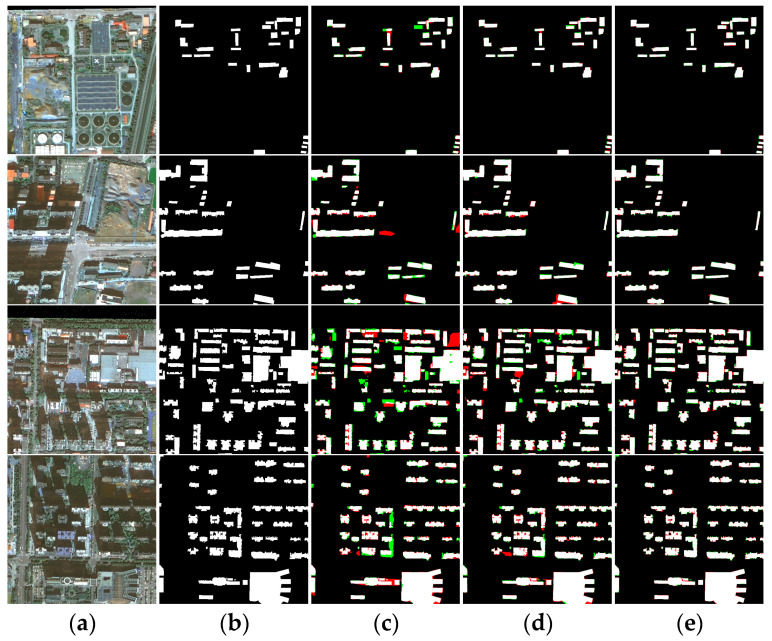
Visualization of SIEM experimental results: (**a**) original images; (**b**) ground truth; (**c**) UNetFormer; (**d**) MARS-Net; (**e**) MARS-Net + SIEM. Colors assigned: TP in white, TN in black, FP in red, and FN in green.

The effectiveness and generalization of MARS-Net have been validated through the experiments above, which further enhance the semantic segmentation performance of the network by exploring the spectral information of images. In this section, experimental validation of the proposed SIEM is conducted based on the MARS-Net network. SIEM uses the spectral information of images’ R, G, B, and NIR bands. Therefore, we used the Xi’an building dataset images for spectral enhancement with SIEM and conducted semantic segmentation experiments with MARS-Net. The visualized results of the prediction are shown in Figure 13e. Table 5 shows the accuracy evaluation results.

In Figure 13, a comparison is made between the MARS-Net network that performed better in the previous experiments and the MARS-Net with the added SIEM module. It can be observed that MARS-Net performs better in predicting sparse small buildings in the first and second rows, but misclassification still occurs occasionally. However, after introducing SIEM, the network can extract building boundary information that closely aligns with the ground truth. In addition, the images in the third and fourth rows contain similar spectral information, complex background details, and dense mixed multi-scale clusters of buildings, leading to less satisfactory extraction results from MARS-Net, with frequent misclassifications and omissions. However, through further processing with SIEM, the misclassifications and omissions in the predicted results are alleviated, and the building information extracted using the network segmentation more closely matches the building forms in the ground truth.

Table 5 shows the evaluation results of the networks. The MARS-Net network with the addition of SIEM achieved improved accuracy evaluation results, with OA, Kappa, IoU, recall, precision, and F1 score reaching 98.20%, 93.04%, 88.86%, 94.21%, 93.99%, and 94.10%, respectively. Compared to MARS-Net, there were improvements of 0.23%, 0.89%, 1.34%, 0.54%, 0.97%, and 0.76% in Kappa, IoU, recall, precision, and F1 score, respectively. It is, therefore, evident that SIEM can effectively enhance the network’s performance.

## 4. Discussion

The scale and morphology of buildings vary due to natural, cultural, and social development. At the same time, the segmentation of buildings is also affected by the different image resolutions and label quality of the dataset, leading to a certain degree of fluctuation in the segmentation results.

By analyzing the characteristics of the two experimental datasets, the WHU aerial buildings dataset has a relatively higher image resolution than the Xi’an satellite buildings dataset. There is a rather large proportion of small buildings in both datasets. However, due to geographic variation, small buildings in the WHU dataset tend to be irregularly shaped like squares, as shown in the first three images in Figure 11. In contrast, small buildings in the Xi’an dataset tend to be long strips and more densely distributed, as shown in the first three images in Figure 10. Influenced by the characteristics of the dataset itself above, in the accuracy evaluation results of the comparison experiments, the training accuracy of each model on the WHU dataset is generally better than that on the Xi’an dataset. As shown in Table 1 and Table 2, ResUNet++, which has a deeper structure, performs lower than U-Net on the Xi’an dataset with poor stability compared to other DCNN networks, although it has higher accuracy. On the two experimental datasets in this paper, the accuracy of transformer class networks is evaluated more elevated than that of DCNN networks, and the performance is more stable. Among them, the MARS-Net accuracy proposed in this paper performs better. Based on the characteristics of the two experimental datasets analyzed in the previous section, this may result from the fact that the building patterns in the Xi’an dataset are mostly long strips. This building morphology gives better play to the transformer’s long-distance extraction advantage. It also enables the transformer-like network to maintain a better extraction effect on the Xi’an dataset, which has a slightly lower image resolution.

In addition, compared with the networks in the other experiments, MARS-Net also has a better segmentation effect on the densely distributed buildings in the two experimental data, as shown in Figure 10 and Figure 11. From the perspective of multi-scale analysis, the richness of spatial and semantic information varies between deep and shallow layers in the feature learning process of the networks. Shallow features often contain more spatial information, but due to their limited depth of learning, they need more semantic feature information. On the other hand, due to multiple rounds of object feature learning, deep features have more accurate semantic feature information. However, after numerous downsamplings, there is some loss of spatial location information, leading to misclassification and omission of detailed information, such as boundaries, corners, and interiors, for small buildings and dense complexes. The MARS-Net proposed in this paper combines the advantages of local extraction from DCNN and global learning from the transformer. It bridges the encoder and decoder at different positions using the CA and CBAM modules to reconcile the contradiction between deep and shallow feature information acquisition. It effectively collects spatial features from the superficial layers and semantic features from the deep layers. At the same time, DenseASPP is used to increase the receptive field during the feature map restoration process in the decoder to obtain semantic feature information at a larger scale. These structures play an essential role in the network and make the network in this paper more effective in segmenting buildings of various scales, forms, and distributions.

The land cover information in high-resolution RSIs is intricate, containing redundant object details. And the similarity in appearance between roads and rooftops significantly affects the interpretation of buildings. However, high-resolution RSIs also have a high spectral resolution. By mining their spectral information, this paper proposes the SIEM to process the maximum weight ratio results of each image band with MBI to enhance the spectral information of the image and further improve the network’s segmentation effect on buildings. We also considered fusing the high-weight ratio bands with MNF at the final stage of SIEM and then stacking the result with MBI to assess accuracy in MARS-Net, as shown in the last row of Table 6. In Table 6, the SIEM in this paper offers a more significant performance improvement than the test results in the previous row. This enhancement may be because the SIEM in this paper fuses the building morphological feature information from the MBI in all bands.

While the proposed method in this paper has achieved good results in building segmentation, there are still some limitations. For example, influenced by geographical location, culture, and the level of social development, the land object information in high-resolution RSIs varies to different extents in different regions. Therefore, spectral information enhancement methods may also need to be adjusted accordingly. We quantitatively compared the performance parameters FLOPs and Params of the experimental network in this paper. As shown in Table 7, the two parameters of MARS-Net are generally better than those of U-Net and ResUnet++, but higher than those of Swin-Unet and UNetFormer. A further comparison of Table 7 reveals that the two performance parameters of the Baseline network structure are very close to the network we proposed. It is speculated that this may be due to our simultaneous use of ResNet and Swin transformer structures to construct a parallel encoder, leading to MARS-Net having higher performance parameters in terms of FLOPs and Params. In future research, we will further adjust and lighten the parallel encoding structure used in the proposed network, continuing to explore universal spectral enhancement methods to adapt to the application scenarios of various high-resolution RSIs. At the same time, according to the spectrally enhanced image features, we will carry out targeted model improvement and design. For example, by better combining the semantic segmentation model and image spectral enhancement method, we may improve the model’s anti-interference ability against phenomena such as shadows and similar spectral features of ground objects and enhance the building segmentation capability of the network more comprehensively.

## 5. Conclusions

The existing building extraction methods often suffer from the complexity of land cover information and the diverse shapes and distributions of buildings, leading to segmentation results that exhibit similarities and adjacency between buildings and surrounding roads. Moreover, incomplete and inaccurate internal and boundary information extraction within buildings is also an issue. First, we constructed the Xi’an building dataset based on the imagery within the third ring road of Xi’an, Shaanxi Province, China, which possesses the characteristic features of local buildings. Due to variations in building shapes, distributions, and image resolutions, this dataset presents more challenges for semantic segmentation networks. Secondly, we propose a deep learning network called MARS-Net, which uses ResNet50 and Swin-T to construct parallel encoders, leveraging the local profound feature extraction advantages of DCNN and the transformer’s global contextual feature extraction advantages. CA and CBAM modules were introduced at different network depths to bridge the encoder and decoder, thus preserving richer spatial and semantic feature information of the building. The DenseASPP module was added during the decoding process to enhance the network’s ability to extract multi-scale building edge features. Beyond that, we designed the SIEM module, which enhances the spectral information of the images by processing the MBI and band ratio results calculated from the R, G, B, and NIR bands in the RSIs, further improving the network’s segmentation accuracy of buildings. Ultimately, we conducted performance analysis experiments on MARS-Net and SIEM. Through comparative experiments and ablation experiments with U-Net, ResUNet++, Deeplab v3+, Swin-Unet, and UNetFormer on the Xi’an building dataset and the WHU building dataset, the results show that our MARS-Net achieves better multi-scale building segmentation performance with different distribution characteristics, stronger resistance to interference from similar spectral features, and higher accuracy evaluation metrics. By processing the Xi’an building dataset images with the SIEM module and continuing the experiments with MARS-Net, the results show that the MARS-Net with the SIEM module is more effective in extracting multi-scale building cluster information with various phenomena, such as similar spectral information and complex background, resulting in clearer boundaries and further improvement in different accuracy evaluation metrics.

## Figures and Tables

**Table 1:** Quantitative evaluation results of comparison networks on Xi’an building dataset (unit: %).

Method	OA	Kappa	IoU	Recall	Precision	F1 Score
U-Net	97.03	88.48	82.20	90.00	90.47	90.23
ResUnet++	96.78	87.56	80.93	89.85	89.07	89.46
Deeplab v3+	95.92	84.38	76.66	87.93	85.67	86.79
Swin-Unet	97.10	88.77	82.62	90.36	90.60	90.48
UNetFormer	97.36	89.78	84.06	91.43	91.24	91.34
MARS-Net	97.97	92.15	87.52	93.67	93.02	93.34

**Table 2:** Quantitative evaluation results of comparison networks on WHU building dataset (unit: %).

Method	OA	Kappa	IoU	Recall	Precision	F1 Score
U-Net	97.66	88.24	81.09	90.39	88.75	89.56
ResUnet++	97.92	89.59	83.08	91.91	89.64	90.76
Deeplab v3+	97.93	89.50	82.92	90.25	91.08	90.66
Swin-Unet	98.22	90.97	85.13	91.52	92.41	91.97
UNetFormer	98.29	91.51	86.01	93.17	91.80	92.48
MARS-Net	98.78	93.84	89.62	94.64	94.41	94.52

**Table 3:** Quantitative evaluation results of ablation experiments on Xi’an building dataset (unit: %).

Method	OA	Kappa	IoU	Recall	Precision	F1 Score
Baseline	96.95	89.03	83.26	93.41	88.46	90.86
Baseline + CA	97.71	91.14	86.02	93.27	91.72	92.49
Baseline + CBAM	97.92	91.95	87.23	93.46	92.90	93.18
Baseline + DenseASPP	97.95	92.09	87.44	93.83	92.77	93.30
MARS-Net	97.97	92.15	87.52	93.67	93.02	93.34

**Table 4:** Quantitative evaluation results of ablation experiments on WHU building dataset (unit: %).

Method	OA	Kappa	IoU	Recall	Precision	F1 Score
Baseline	98.39	91.73	86.27	91.72	93.55	92.63
Baseline + CA	98.65	93.18	88.57	94.12	93.77	93.94
Baseline + CBAM	98.68	93.30	88.76	94.01	94.08	94.04
Baseline + DenseASPP	98.70	93.45	89.00	94.51	93.85	94.18
MARS-Net	98.78	93.84	89.62	94.64	94.41	94.52

**Table 5:** Quantitative evaluation results of SIEM module on Xi’an building dataset (unit: %).

Method	OA	Kappa	IoU	Recall	Precision	F1 Score
MARS-Net	97.97	92.15	87.52	93.67	93.02	93.34
MARS-Net + SIEM	98.20	93.04	88.86	94.21	93.99	94.10

**Table 6:** Quantitative evaluation results of test experiments on Xi’an building dataset (unit: %).

Method	OA	Kappa	IoU	Recall	Precision	F1 Score
MARS-Net	97.97	92.15	87.52	93.67	93.02	93.34
MARS-Net + SIEM	98.20	93.04	88.86	94.21	93.99	94.10
Test	98.06	92.48	88.02	94.05	93.21	93.63

**Table 7:** Comparative results of performance parameters for the networks.

Method	FLOPs (G)	Params (M)
U-Net	3593.30	65.85
ResUnet++	8186.80	352.72
DeepLabV3+	590.83	22.18
Swin-Unet	691.16	103.55
UNetFormer	262.36	44.56
MARS-Net	1002.46	203.22
Baseline	999.69	202.96

## Data Availability

The data presented in this study are available on request from the corresponding author.
